# School Nurses’ Perceptions About Student's Wellbeing During the Covid-19 Pandemic in Sweden

**DOI:** 10.1177/10598405221112443

**Published:** 2022-07-12

**Authors:** Eva Martinsson, Pernilla Garmy, Eva-Lena Einberg

**Affiliations:** 1 Faculty of Health Sciences, 4342 Kristianstad University, Kristianstad, Sweden; 2 Department of Health Sciences, Faculty of Medicine, Lund University, Lund, Sweden

**Keywords:** adolescents, Covid-19, focus group, school nurse, school children, well-being, perceptions, students

## Abstract

The coronavirus 2019 (Covid-19) pandemic has affected both the private and public lives of people worldwide. Countries have chosen different strategies to reduce the spread of infection, including school closures and distance learning. This study aimed to describe school nurses’ perceptions about the wellbeing of students during the Covid-19 pandemic in Sweden. Interviews in five focus groups and one individual were conducted with 17 school nurses in Sweden. The interviews were analyzed using a qualitative content analysis. According to the school nurses’ perceptions, students were concerned about spreading infection, becoming infected themselves, their academic performances, and longing for socialization. The change in the school situation involved a slower pace and less anxiety for some students, more or less physical activity, and an increase in screen time.

The coronavirus 2019 (Covid-19) pandemic is a societal crisis that has affected the private and public lives of people at both local and global levels. The pandemic has contributed to an increase in psychological distress in the form of stress, anxiety, worry, and loneliness in children and adolescents (Andersson et al., 2021; Brooks et al., 2020; Liu et al., 2021; Wang et al., 2020). Students and school staff have been affected to a great extent. Countries have chosen different strategies to reduce the spread of infection. Many have chosen lock downs (a shutdown of the entire society). On March 18, 2020, one-hundred seven countries chose national school closure, which affected approximately 862 million children and young people in these counties and corresponds to half the world's students (Viner et al., 2020). In the United Nations (2020) report from August 2020, 1,600 million children in 190 countries were affected.

Lock down meant different levels of isolation for different lengths of time, which affected mental health to a large extent for both younger and older children (Holmberg, 2020; McCracken et al., 2020). School closures and distance learning affected the students both negatively and positively. In a review article involving 24 studies, several negative psychological effects were highlighted, such as post-traumatic stress (PTS), confusion, and anger. The stressors were longer quarantine durations, infection fear frustrations, boredom, inadequate supplies, financial loss, stigma due to quarantine, and inadequate information about the pandemic (Brooks et al., 2020). Children and young people get their information about Covid 19 from different sources. A study from the United Kingdom (UK), Brazil, Spain, Canada, Australia, and Sweden showed that in all countries except Sweden, children between the ages of 7 and 12 usually received information about Covid-19 via their parents, while in Sweden, the schools were the main source of information about Covid-19 (Bray et al., 2021). One reason for this difference may be that Sweden chose to keep the schools open during the pandemic (Lindblad et al., 2021). Swedish students have been asked about their experiences concerning the Covid-19 situation. A study by Sarkadi et al. (2021) focused on expressions of worry in children ranging in age from 4 to 18 years old during Covid-19. Worry was a common any redisease or death of elderly relatives, parents, the child him/herself and/or general worry for the elderly/high-risk groups. An interview study by Hörbo et al. (2021) shows that adolescents (aged 13–15 years) were relatively unaffected by the pandemic although concerns about relatives who could get sick and how the future will look, such as possibilities to hug older relatives and go to festivals and abroad. Furthermore, a survey by Chen et al. (2021) showed that no difference in mental health status changes, relationships with parents and peers, and health habits during 2020 between those who have had Covid-19 and those who have not in changes in mental health was found.

Countries have different solutions for distance education in which some students received instruction via radio or TV, others were allowed to work on paper, and in several countries, instructions were given via the Internet and different communication platforms. In Sweden, a few studies about the readiness to quickly switch to distance education and how the students managed the transition have been published (Åkerfeldt & Hermansson, 2021; Lidegran et al., 2021). Lidegran et al. (2021) highlights three groups who have different problems during distance learning: (1) Upper- and middle-class youth who had problems with the boundary between school and leisure time, (2) Adolescents in rural areas who had difficulty maintaining their studies, took naps, and took it easy, and (3) immigrant adolescents in the working class who did not get the support and instructions that they thought they needed. Åkerfeldt and Hermansson’s (2021) reported on distance learning for which 2,300 students responded and revealed a complex picture in which many see both the pros and cons of distance learning and on-site teaching. However, most people preferred to be in school, but 20% liked distance learning. Students thought that remote teaching worked relatively well and the teachers were engaged, but it was more difficult to get help. Other studies showed that often already vulnerable groups performed the worse during this period (Åstrand, 2020; Garcia de Avila et al., 2020). The school environment itself is a protective factor for young people's mental well-being as several studies have shown (Granvik Saminathen et al., 2020; Wilhsson et al., 2017).

In Sweden, the elementary schools with students ranging in age from 6 to 15 years old were open and had students on site throughout 2020. The upper secondary schools with students ranging in age from 16 to 19 years used remote teaching from March 18, 2020 until the spring term (January to June) ended. During the autumn term (August to December of 2020), the upper secondary school students were on site, but in some schools, they switched between on site and distance learning only to switch to distance learning only again in December. In elementary schools, the Swedish Schools Inspectorate's review 2020 revealed that an increase in student and staff absenteeism in the spring of 2020 had occurred. The principals who were interviewed reported that they did not see any major loss of knowledge among the students at that time. The students who were interviewed stated that no widespread concern about the pandemic among the students existed and that they often knew to whom to turn if they felt worried. At the same time, students called for more proactive and outreach work on the part of the school to identify and support the students who experienced anxiety (The Swedish Schools Inspectorate, 2020). The Swedish Schools Inspectorate's review 2021 of distance education revealed that particularly vulnerable groups were students in preparatory classes, namely those with failed school courses, immigrants whose second language was Swedish, and students with social anxiety at home. The mental and physical health of many students was negatively affected by distance learning. Stress, loneliness, and poor living habits emerged during the pandemic (The Swedish Schools Inspectorate, 2021).

International research and debates show that the school nurses in many countries played an important role in students’ well-being during the pandemic and in many cases, even during school closures (McDonald, 2020; Rosario, 2020; Rothstein, & Olympia, 2020). The school nurse is an important public healthcare worker in the Swedish context. The competence descriptions for school nurses state that the school nurse should primarily work with promoting physical health and preventing mental illness, reduce risk factors, and strengthen protective factors for the student (Swedish Association of School Nurses and Swedish Society of Nursing, 2016). The school nurse's work with health conversations can both be preventive and can also reveal those who need more support (Berglund Melendez et al., 2020, Ellertsson et al., 2017; Golsäter et al., 2010; Rising Holmström & Boström, 2021). Martinsson et al. (2021) highlighted the school nurses’ work in Sweden during the first year of Covid-19. These authors emphasize that school nurses prioritize students’ needs and adapt their working methods to support students based on conditions that are constantly changing. However, to our knowledge, no studies in the Swedish context that focus on the school nurses view of the pupils’ wellbeing are available. Therefore, this study aimed to describe school nurses’ perceptions about the wellbeing of students during the Covid-19 pandemic in Sweden.

## Methods

### Design

An explorative qualitative study design was used (Polit & Beck, 2021). Interviews with five focus groups and one individual interview session was performed between the end of November 2020 and January 2021 in Sweden. The study was approved by the Regional Ethics Review Board in Lund, Sweden (EPN 2018/842; 2020-04871).

### Participants and Setting

Purposive sampling (Polit & Beck, 2021) was used and finally yielded a sample with 17 school nurses working with students aged 6–19 in Sweden throughout 2020. The school nurses work work-related experience ranged from 1.5 to 20 years, and the nurses were all women. Eight school nurses, distributed between two focus groups and individual interviews, worked in elementary schools (students aged 6–15 years), and nine school nurses, distributed between three focus groups, worked in upper secondary schools (students aged 16–19 years) as shown in Table 1. Variations were obtained when both private and public schools and schools from rural and urban areas in addition to schools from different socio-economic contexts were represented. Furthermore, municipalities with low and high prevalence of inhabitants with Covid-19 during the data collection period were selected.

**Table 1.** Focus Groups and Individual Interviews, Number of Participants, and Workplaces.

Focus group/interview (Number)	Participants (n)	Workplaces
1	4	Elementary school (students aged 6–15 years)
2	3	Upper secondary school (students aged 16–19 years)
3	3	Elementary school (students aged 6–15 years)
4	2	Upper secondary school (students aged 16–19 years)
5	4	Upper secondary school (students aged 16–19 years)
6	1	Elementary school (students aged 6–15 years)

### Data Collection

Information about the study was sent to the school health administration in 12 municipalities. Six of them agreed to participate, and in these municipalities, 16 school nurses expressed interest in participating. Information about the study was also sent to a manager in a private company for Swedish school nurses, resulting in another three school nurses who agreed to participate. In total, 19 school nurses gave written informed consent to participate. The focus groups were designed to consist of 3 to 4 school nurses and were related to the researcher's experiences with digital solutions and meetings. When meeting digitally, more than 4–5 participants complicated the dialogue and the opportunity to create a discussion between the participants. In this situation, the discussion between the participants tended to be absent. However, on short notice, two participants were prevented from participating, and therefore one focus group consisted of two participants, and one focus group became an individual interview. Since all interviews, consisting of one individual interview and five focus groups, provided valuable data, all were included in the analysis.

The interviews were conducted in a semi-structured format via Zoom, a digital meeting application. An interview guide was used and evaluated by the first and last author after the first focus group interview was performed. In addition to some minor changes regarding the order in which the questions would be asked, the interview guide was perceived to work well. Examples of questions in the interview guide: “*What do your students do to feel good? Has it changed during the pandemic?*” In all five focus group interviews, the first author acted as a moderator and the last author as an observer. The first author performed the individual interview. The individual interview took about 45 min, and each focus group interview took about one hour.

### Data Analysis

The focus group interviews were transcribed verbatim and then analyzed using inductive content analysis according to Elo and Kyngäs (2008). First, each focus group interview transcript was read thoroughly several times to become familiar with the content. When reading through the transcript notes was done in the margins, this process was termed open coding. To organize the data, all meaningful units with relevance to the aim of the study were removed and transferred to separate documents and to facilitate the analysis of the meaning units, they were then condensed and coded (cf. Table 2). This initial analysis was conducted by the first author. Thereafter, all authors met to discuss and identify similarities and differences in addition to creating categories. The process of analysis went back and forth, including re-listening to the recorded material and re-reading transcribed interviews. After discussions between all authors, consensus was reached. For an overview of the analysis process, see Table 2.

**Table 2.** An Overview of the Analysis Process.

Meaning Units	Condensed	Coded	Categorization
Chronically ill parents or elderly parents that students are afraid of infecting (1)	Afraid of infecting relatives	Spread the infection	Concerns about spreading infection
The students quickly became very concerned this spring at my school. They became very vocal at school, “who is contagious and who is not contagious?” (3)	Concerned over who is contagious	Become infected	Worry about becoming infected yourself
The students say, “I will never have time to get these grades”. There is a lot of concern about schooling. (2)	Concerns about schooling and grades	Performance and grades	Concerns about academic performance
Some have a lot of social anxiety and find it difficult to go and eat in the dining room. It was better for them to study from home because they got rid of that stress. (4)	Study from home gives less anxiety	Less anxiety	Slower pace and less anxiety
Chat groups have been created throughout the class. This means that you are not forgotten if you are away for a long time. (3)	Chat groups to not be forgotten	Meet classmates	Longing for socializing
They have started going out for walks a lot more. (5) Physical activity, it has been on the downside. (6)	They walk more Less physical activity	Physical activity	Physical activity more or less
I can agree with those you unwind and calm down and forget all problems through series and play games. (5)	Series and games to forgot problems	Screen time	Increased screen time for better or worse

## Results

The analysis of the collected material resulted in formation of seven categories that describe perceptions of school nurses about students’ wellbeing during the Covid-19 pandemic in Sweden. The school nurses’ narratives indicate that students worried about spreading the infection to relatives, and some also worried about becoming infected themselves. According to the school nurses, the change in the school situation caused stress over academic performance and the future, especially for the older students, and they reported that they missed socializing with friends. On other hand, for some students, the change in the school situation meant a slower pace and less anxiety. The school nurses’ narratives show that students’ physical activity both decreased and increased. Differences between students at elementary school and upper secondary school and between younger and older students in elementary school indicated different levels of physical activities depending on age group.

Seven categories are presented: (1) concerns about spreading infection, (2) worry about becoming infected yourself, (3) concerns about academic performance, (4) slower pace and less anxiety for some, (5) the longing for socialization, (6) more or less physical activity, and (7) an increase in screen time for better or worse. Quotes from school nurses were numbered according to each focus group (1–5) and individual interviews (6).

### Concerns About Spreading Infection

The school nurses said that many children and adolescents were worried about spreading the infection. These students may have had older parents or at-risk family members. They may also have worried about spreading the infection to their grandparents. The consequence of this fear was that many students stayed home from school even when the schools were open. Some students were also kept at home by their parents.

At home, there was much talk about the coronavirus and its consequences, and that mothers and grandparents can end up in the hospital and may die. (1)

This is another concern with family and relatives and those at risk. There is a lot of spread at my school—both among staff and students. It's getting tough. (3)

They were afraid of exposing their family members to infection and stayed at home most of the time. (5)

### Worry About Becoming Infected Yourself

The school nurses described children and adolescents who had been worried about falling ill with Covid-19 themselves. The school nurses said that some students followed the news very closely, and a few students panicked after they heard and read about the virus. The school nurses also described students who did not dare to go out for fear of becoming infected. In some schools, it became very noisy as the students became worried about who was contagious.

Children have panicked. They do not want to come to school for fear of getting it [Covid-19]. One student was manically [extremely] scared; the student was completely absorbed and constantly watched the news. (1)

This stems from a worry of getting sick yourself. (2)

The students quickly became very concerned this spring at my school. They became very vocal at school, “who is contagious and who is not contagious?” (3)

Many of my students stayed at home and did not dare to go out. We tried to encourage them to go out—that nothing would happen. That was the case this spring, but no, they were afraid [of being infected]. (5)

### Concerns About Academic Performance

The school nurses described the stress and anxiety among the students regarding their academic performances caused by the pandemic. Homeschooling created stress for many students. In addition, several national exams were canceled during the pandemic. The school nurses said that many students felt extremely stressed because they felt that their future depended on how well they performed in school. Students in elementary school in which most on-site teaching occurred were also affected by the pandemic. Higher absenteeism was noted because students and staff would stay home after noticing the slightest symptom, and this process could make students feel stressed about how to cope with school and implied the need for more substitute teachers, which could also have affected the elementary school children's stress and anxiety levels.

The indirect stress for the students /../ because it is a turnover that there are a lot of teacher substitutes. I am thinking of the students who need a lot of structuring. (1)

This stress of not being able to complete your studies. To miss exams and to not be allowed to take national exams. How does it affect my grade? (2)

There are many worries for the future. How is school going? Are they going to make it? How are the grades? (4)

They come and complain that they are not feeling well. They are worried about how to cope with schoolwork. (5)

### Slower pace and less anxiety for some

The school nurses also talked about the positive consequences of the pandemic for some students’ well-being. The pace became a little calmer during the pandemic, and the school nurses described students who thought it was a relief to be able to plan for themselves and have time for hobbies. Students with social anxiety may have had higher attendance levels because the teaching was conducted as distance learning. Avoiding the school canteen and eating at home instead (for the older students) or in the classroom (for the younger ones) was experienced as a relief by some. The school nurses also said that restrictions on meetings in groups was a relief for some students. Some students were calmer when not having to meet friends in a group.

The younger students now eat in the classroom, and they are very happy that they do not have to go to the school canteen. (1)

Some become less stressed by the fact that it is rare to meet in groups. They are at home more, which makes everything a little calmer for many students. (3)

There were several students who experienced it as wonderful and thought it was a relief to be able to plan themselves and maybe get some time for things that they thought were fun. (4)

Some have actually been able to concentrate better when they have been at home and have had less school absenteeism. (5)

### The Longing for Socialization

The school nurses in upper secondary schools highlighted how the students longed for socialization during distance learning. One school nurse gave an example in which she had asked the students to raise a hand if they had talked to somebody today, and only 5 out of 32 raised their hand. The school nurses described the enormous need to talk to the students and the importance of sometimes meeting someone from the school. Those who took vocational courses were allowed to come to school after a while to do practical things at school in small groups. This activity was much appreciated. Some of the students met their friends at home, while others met online. For younger children who went to meet their friends at school every day, the change involved cancellation of their activities and spending more time with their families.

There was an incredible need to talk. (2)

The children feel very good about being able to come to school and everything is almost as usual there anyway. (3)

Everyone misses this social part. (5)

A girl said “I long for June when we can go back to [name of home country] to meet our relatives”. (6)

One way for students in the secondary upper school to meet when teaching was done at a distance was to go and pick up their lunches. Some picked a lunch up at their school or an elementary school near his/her residence or the municipality provided coupons that they could use to shop for their lunches. This lunch period also meant meeting their friends or other students who were picking up food.

We had several gangs who had an outdoor lunch. It was a great way to socialize. (4)

### More or less physical activity

The school nurses expressed that physical activity among students improved in some ways and decreased in others. Many school nurses reported that the younger children played more outdoors during the pandemic compared to before the pandemic and that the older adolescents engaged in more walking. Some school nurses also pointed out that many students continued with their physical activity, and when the indoor training ceased, they trained outdoors instead. However, several school nurses also pointed out that physical activity went down when the organized sports were cancelled.

The students play outside more. (1)

They are out very much in preschool class to grade six. (3)

The students were affected by that sports activities shut down. (5)

I have understood during the health visits, that it is much less organized activity if you think about to maintain your health. It is much less organized sports. (6)

### Increased Screen Time for Better or Worse

The school nurses expressed that many of the students had more screen time when activities were canceled, and they were not allowed to meet friends. However, screen time was different in different age groups. Among the younger ones, more conflicts caused by social media were noted. One school nurse stated that she found that bullying increased at her school when children communicated on social media, such as the digital application, Snapchat. Some upper secondary school classes arranged class chats so they could communicate and have contact with those who were at home for an extended period. In upper secondary school, the school nurses emphasized that screen time was a way for the students to unwind and relax when they watched series and/or YouTube clips and/or played games. This screen time was a way to socialize via social media.

We all have our gaming nerds gaming guys because it is often guys who play them think this is fun time because then they get a reason to sit even more at the computers /… / Because they are in their gaming world and this is where they have a social network in a completely different way, after all. (1)

If chat groups have been created throughout the class, this means that you will not be forgotten if you are away for a long time. (3)

Yes, I absolutely think they find other ways and sometimes much smarter ways than we have devised ourselves. (5)

## Discussion

This study reports school nurses’ perceptions on how the students in the Swedish school were affected by Covid-19. Seven categories were identified in the results. The findings are different for the younger and older students. The results present a nuanced picture. Previous studies show that the findings could be different in different groups and different countries (Bray et al., 2021; Marchi et al., 2021). Fioretti et al. (2020) highlights both positive and negative pandemic-related impacts. The restriction of autonomy was negative for adolescents because they had to be at home, the social part of school disappeared, school became just a knowledge gathering, the routines had changed, and they felt anxiety and missed socializing. Positive feelings included being part of an extraordinary event, discovering oneself, rediscovering his/her family, and sharing life remotely. In our study, students in the elementary school were present at the school when the study was carried out, and the upper secondary school students were engaged in remote learning from March 2020 and were on-site and remote in the autumn of 2020.

Among the older students, in upper secondary school, a greater awareness of spreading infection was reported, and many wanted to protect their family and elderly relatives from infection. Hörbo et al. (2021) notes that adolescents were most worried that their relatives would get sick. Some students, according to the school nurses in our study, also had a concern about becoming ill themselves. Chen et al. (2021) examined the mental health of children in Sweden who have/have not been infected by Covid-19. No difference between the groups were found. In our study, for the younger students, the negative aspect was an increase in absence among the school staff and students due to the restrictions and the infection situation. The younger children, 6–12 years of age, who are very much living in the moment, were not so worried about the infection situation, except for a few cases, but felt worried about changes in their everyday life, such as parents working at home and not being allowed to meet grandparents as Hörbo et al. (2021) also highlighted. This finding was also reported from the children themselves in the study of Sarkadi et al. (2021) in which the children expressed that they were worried that grandparents and others close to them would become infected. Based on findings in current and earlier studies, school nurses have an important role to play by listening and providing reliable information to students to alleviate their concerns.

The findings in our study highlight the longing for social interaction and concerns about academic performance. Those parameters changed the most for older students. Upper secondary students lacked time with their friends and their social context at school when they switched to remote learning. Kapetanovic et al. (2021) reported that 49.6% of students had a reduction in socialization with their friends during the Covid-19 outbreak. For 27.8%, it was unchanged and for some this time represented an increased interaction with a few friends. Other studies have shown that upper secondary school students were also concerned about their study results. Many felt lonely and unable to collaborate with their friends in the same way as when they were in the classroom. Many also lack the routines that teaching in school provides throughout the day as it was easier to distinguish between school and leisure while in school (Åkerfeldt & Hermansson, 2021; Hörbo et al., 2021). School nurses’ unique position provided them with possibilities to capture students in need of support as they meet every students on a regular basis and to collaborate with other school personal support to achieve the same goals.

In our study, it emerged that the school nurses perceived that some students in upper secondary school thought it was beneficial to have distance learning. Two groups stand out: (1) those who were good at planning and had self-discipline and (2) those who did not like being in large groups or had some form of social phobia. Others had difficulty keeping up with the studies, but some adjustments were made so that, for example, practical parts could be carried out at the school in smaller groups. Ahlström et al. (2020) also highlights that the gap in knowledge between different groups of students increased during the distance learning period; the vulnerable groups needed more support because they had fallen behind during this period. This finding has also been described by the Swedish School Inspectorate (2021) and the Swedish National Agency for Education's investigations (Rönnström, 2021). Kapetanovic et al. (2021) reported that most adolescents coped well with psychosocial changes; however, a smaller group had a harder time. Many have less control over their lives and mental health was negatively affected, especially for those who underwent distance learning. Andersson et al. (2021) reported significant negative consequences related to mental health and school performance. Kerekes et al. (2021) highlights a gender gap for which girls felt worse, but the researchers also pointed out that many managed the adjustments to the new situation well. Accordingly, professionals at schools, including school nurses, needed to adjust and offer different forms of support to different groups of students in addition to taking advantage of the positive experience students have reported concerning distance learning students.

Elementary school students met at school every day, except that the absence rate was higher due to the restrictions and socializing with friends in their spare time had decreased. Screen time increased for many children and adolescents for which international studies also has shown (Ghanamah & Eghbaria-Ghanamah, 2021). In our study, the finding that social media provide a way to keep in touch with each other for the older groups of students emerged. Those who were home due to mild symptoms could find out what had happened at school through class chats. A study by Ellis et al. (2020a, 2020b) focused on increasing internet use by adolescents during the pandemic. Those who participated in social media frequently became more depressed than those who spent more time with family and did schoolwork. Having contact with your friends digitally, spending time with family, and physical activity made this other group feel less alone. The younger students increased their screen time due to cancelled leisure activities, which in this age group could lead to more bullying (Ellis et al., 2020a, 2020b). Marchi et al. (2021) also highlighted the possible negative impact of an increased use of internet, computer games, and social media. A review by Viner et al. (2022) pointed out more screentime and less physical activity. Since advantages and disadvantages to internet use as social media can be found, school nurses and teachers need to have a nuanced dialogue with students about this topic.

The school nurses sometimes gave a contradictory picture of the changes in everyday life for the students for whom physical activity and pace of life changed. A Swedish study concerning the adult population shows that 71% had unchanged physical activity, while 29% had reduced theirs (Blom et al., 2021). An American study shows that lack of exercise negatively affected mental health, especially for those who exercise team sports (McGuine et al., 2020). In our study, many upper secondary students continued their outdoor training when indoor workout facilities were closed. Others lost their training and were less physically active. A study by Ellis et al. (2020a, 2020b) with participants from 66 countries who played games, including physical activity such as Pokémon go, shows that physical activity decreased by an average of 7.5 h per week. The restrictions related to the pandemic varied in different countries in terms of the possibility of being outside, which affected the outcome of the study. Some school nurses in current study recommended digital applications, such as Pokémon go, which encouraged physical activity when other activities were cancelled. The change that most affected the younger students of ages ranging from 6 to 12 years of age was a decrease in leisure activities. However, in some schools, the students were outdoors more often, for example, having lunch at school outside and playing with friends outdoors both at school and during leisure time. For many the experience was positive as it became calmer at school and home with more time spent with the family. Several of the school nurses’ observations are consistent with what children and adolescents themselves expressed in studies. A study by Sarkadi et al. (2021) highlighted that among other aspects, younger children appreciated the slower pace and spending more time with the family. Vira and Skoog’s (2021) longitudinal study concerning middle school students reported that these students adapted well to the pandemic situation, and their wellbeing remained the same as before the pandemic with no significant differences. Findings from current and earlier studies show a nuanced picture of experiences related to Covid-19. School nurses in addition to other school professionals may use this knowledge in their work to promote students’ health and development.

## Strengths and Limitations

It can be argued that it would be beneficial to interview students directly about their experiences according to the Covid-19. However, different perspectives may give a broader picture of the situation; therefore, the current study complements studies that have reported student perspectives. A strength of this study is that additional thoughts emerged in more than one focus group, which increased the trustworthiness of the results (Polit & Beck, 2021). Furthermore, in the focus groups, in addition to the moderator, an observer who could summarize and collect threads to be discussed at the end of the focus group was also present. A limitation may be that one focus group consisted only of two school nurses, and one interview was individual because of dropouts on short notice. Nevertheless, these two interviews provided valuable material as did the other focus groups with three to four participants, which is the reason that all were included in the analysis. Another limitation may be that the focus groups had to be conducted digitally due to the pandemic, which could have affected the discussions. However, the participating school nurses did not seem to be restricted by the digital form, and in the end, the narratives were valuable. At last, the transferability of the results may be restricted due to the Swedish school context.

## Conclusions

According to the school nurses’ perceptions, the wellbeing of students during the Covid-19 pandemic in Sweden was affected in different ways and related to the students ages and school forms. The school nurses reported that students expressed concern about spreading infection to relatives, and some also worried about becoming infected themselves. The students expressed concern about academic performance, how it can affect their future, and the longing for socialization. The restrictions related to the pandemic and the change in the school situation also meant slower pace and less anxiety for some students. Physical activity decreased for some students and increased for others, and according to the school nurses, increase in screen time provided both positive and negative effects.

## Implications for School Nursing and Future Research

School nurses have a unique position at schools as they work close to both students and the school staff. The Education Act (2010) states that students should have school health services, access to a school nurse, and be offered recurring health visits. Accordingly, school nurses can capture different needs of students in situations, such as a pandemic and subsequent restrictions. Our study shows that school nurses contributed a more nuanced picture of students’ well-being, which can provide valuable knowledge in planning suitable interventions in circumstances, such as a pandemic. The school nurse's unique competence in relation to the school context contributes to the school's mission to offer a good study environment, including promotion of the health and development of students (Swedish Association of School Nurses and Swedish Society of Nursing, 2016). Based on the results of this study school nurses need to pay attention to vulnerable groups so these groups get the support they need. If students are worried, the school nurse is a resource who can provide reliable information but also offer support by being available and listening. School nurses have medical knowledge unlike other school staff and therefore can explain information related to infections and subsequent restrictions to students and also to school staff. Furthermore, school nurses may participate in health dialogue and other encounters with students and detect students early who need extra support to manage school in case of distance learning. School nurses may use the results in this study to discuss with other professionals at school how they can learn from what has emerged as something positive due to restrictions related to the pandemic, such as increased physical activity among the younger school children.

The need for more research about school nurses’ contributions to supporting student health during events, such as the pandemic or other large societal changes, exists. Research looking at different school contexts and organizations and how they affect school nurses’ possibilities to support students should be undertaken. This knowledge may lay the foundation for helping to better meet students’ need in case of future events in the society.
